# Altered frequency architecture of spontaneous brain activity in asymptomatic carotid stenosis: a wavelet-based resting-state fMRI study

**DOI:** 10.3389/fneur.2026.1683526

**Published:** 2026-01-22

**Authors:** Wei Xue, Zongyuan Qin, Xiaoli Zhong, Liangliang An, Hanming Shi, Haifeng Wang, Jinjun Wang, Lei Gao, Yang Zhao

**Affiliations:** 1 Department of Radiology, The Second Hospital of Tianjin Medical University, Tianjin, China; 2 Department of Radiology, Yuncheng Central Hospital Affiliated to Shanxi Medical University, Yuncheng, China; 3 Department of Radiology, Zhongnan Hospital of Wuhan University, Wuhan, China; 4 Department of Ultrasound, Yuncheng Central Hospital Affiliated to Shanxi Medical University, Yuncheng, China; 5 Department of Radiology, Lanxi People's Hospital, Lanxi, China

**Keywords:** cortical hierarchy, cross-frequency coupling, default mode network, vascular cognitive impairment, wavelet-ALFF

## Abstract

The intrinsic brain activity measured by resting-state fMRI (rs-fMRI) consists of synchronized neural oscillations across a broad range of low frequencies. Although previous studies have linked frequency-specific changes to cognitive function and impairment, the alterations of these frequency-specific spatiotemporal patterns in chronic occlusive cerebrovascular disease remain unclear. In this study, we investigated the cross-frequency structure underlying cognitive impairment in patients with severe asymptomatic carotid stenosis (SACS) using wavelet-transformed amplitude of low-frequency fluctuation (wavelet-ALFF) of rs-fMRI. We found that, in healthy controls, frequency-specific wavelet-ALFF exhibited a spatial distribution from lower to higher frequencies, aligned with the functional hierarchy extending from the default mode network (DMN) to primary somatomotor and subcortical regions. In contrast, SACS patients exhibited frequency-dependent changes, including significantly decreased wavelet-ALFF in the anteromedial DMN at lower frequencies and the posteromedial DMN at higher frequencies. Further spatiotemporal decomposition analysis revealed that SACS patients exhibited abnormal cross-frequency coupling in the DMN. Our findings suggest that frequency-specific changes underlying cognitive impairment in SACS arise from spatiotemporally abnormal cross-frequency interplay within the DMN. These insights may contribute to a better understanding of other major brain diseases.

## 1 Introduction

Carotid stenosis is a well-known contributor to ischemic stroke and serves as an independent risk factor for vascular dementia. Over the past two decades, the management of carotid stenosis has evolved from predominantly aggressive endovascular interventions to a focus on intensive medical therapy, leading to a reduction in the annual stroke rate to below 1% (1). This shift underscores the importance of identifying high-risk individuals and incorporating cognitive status as a key monitoring indicator. Growing evidence suggests that even clinically “asymptomatic” carotid stenosis is associated with multidomain cognitive impairment (2–6). Yet the functional brain mechanisms underlying cognitive impairment in patients with carotid stenosis remain poorly understood.

The functional basis of human cognition depends on spatiotemporally organized oscillatory activity, which can be measured using resting-state functional magnetic resonance imaging (rs-fMRI) (7, 8). By examining low frequency (typically < 0.1 Hz) blood-oxygen-level-dependent (BOLD) signals, rs-fMRI has revealed important organizational features of intrinsic brain dynamics, including large-scale networks, the functional cortical hierarchy, and frequency-specific local fluctuation oscillations (LFOs) (8–12).

These investigations have greatly advanced our understanding of brain aging and cognitive processes. Yet, most discoveries in this field have relied on a uniform low-frequency framework, typically < 0.1 Hz. A growing body of evidence suggests that LFOs in rs-fMRI signals may also exhibit an organized structure along the frequency dimension, sensitive to both physiological and pathological conditions (10, 13–17).

Documented evidence indicates that many pathological processes involve regionally distinct and frequency-dependent alterations in LFOs. For example, amnestic mild cognitive impairment (MCI) shows significant alterations in the default mode network (DMN), characterized by dominant lower frequency (slow-5: 0.01–0.027 Hz) activity (18), whereas schizophrenia and chronic pain exhibit alterations in sensorimotor processing circuits at higher (0.12–0.20 Hz) frequencies (19, 20). The DMN and sensorimotor systems represent opposite ends of a hierarchical structure in the human brain (11). Specifically, the DMN, situated at the top of this hierarchy and distant from primary sensory and motor cortices, is involved in relatively slow forms of information processing, such as memory consolidation, autobiographical memory, and social cognition [for example, see (21)]. In contrast, the primary sensory and motor cortices, together with certain subcortical regions, reside at the lower end of this structure and primarily respond to immediate sensory inputs and environmental stimuli (22–24). Other frequency-dependent analyses have further suggested that communication between frequency bands plays a critical role in neural coordination, with low-frequency oscillations modulating high-frequency activity and high-frequency dynamics providing feedback to lower frequencies (17, 25–27). Taken together, these observations suggest that frequency-specific abnormalities may stem from spatial reorganization or disrupted cross-frequency coupling.

Cognitive impairment associated with carotid stenosis has been linked to dysfunction of LFOs in both the temporal and frequency domains. Previous studies have reported altered functional connectivity within the DMN over time (28, 29), as well as decreased amplitude along with frequency-specific changes in LFOs (30) among individuals with carotid stenosis. Moreover, patients with carotid stenosis have been showed to exhibit partial or even complete reversal of global cognitive impairment after revascularization, accompanied by increases in cerebral blood flow and LFOs within the DMN (31), suggesting that DMN oscillations may serve as a potential neuroimaging marker of cognitive restoration. However, the extent to which carotid stenosis affects the frequency structure of the DMN remains unclear.

Notably, potential pathologies associated with carotid stenosis include prolonged cerebral hemodynamic latency, chronic hypoperfusion, microinfarcts, and structural dysconnectivity (32). These pathophysiological changes can lead to hemodynamic delays, reduced amplitude, and altered functional coherence of LFOs. For example, evidence from non-disabling stroke cohorts suggests that a hemodynamic delay of approximately 4 seconds persists on the affected hemisphere even one year after stroke (33). Chronic hypoperfusion and cortical microinfarcts have been associated with impaired neurovascular coupling and gray matter loss (34). Furthermore, white matter hyperintensity (WMH) has been shown to disrupt both cost-efficiency balance and hub-node configuration in large-scale functional networks (35). Taken together, these findings suggest that the pathological consequences of carotid stenosis may disrupt frequency organization and interfere with cross-frequency coupling of resting-state BOLD signals. Nevertheless, studies explicitly investigating frequency reorganization in cognitive impairment related to carotid stenosis remain scarce.

To address this gap, the present study examined potential alterations in frequency reorganization among individuals with unilateral severe (>70% narrowing) asymptomatic carotid stenosis (SACS) using resting-state fMRI (rs-fMRI). While most rs-fMRI studies have concentrated on temporal dimensions (i.e., dynamic interactions), the frequency dimension has received far less attention. Specifically, we employed an updated version of wavelet transformation of amplitude of low-frequency fluctuation (wavelet-ALFF), which effectively characterizes signals in both temporal and frequency domains. We hypothesized that frequency-specific alterations in SACS patients can be decomposed into changes in spatially dominant distribution and temporally altered cross-frequency coupling. These abnormalities would involve the DMN at lower frequencies and somatosensory/motor cortices at higher frequencies, and are closely associated with WMH lesions and gray matter atrophy.

## 2 Materials and methods

### 2.1 Participants

Thirty SACS patients were recruited, and the inclusion criteria were (a) 55–80 years; (b) unilateral internal carotid artery (ICA) stenosis ≥ 70%; (c) right-handed; (d) free of stroke, transient ischemic attack, or dementia; (e) Modified Rankin Scale (36) score of 0 or 1. Exclusion criteria were (a) contralateral ICA stenosis ≥ 50%; (b) posterior circulation diseases; (c) Mini-Mental State Examination (MMSE) (37) score < 26; (d) functional disability (Modified Rankin Scale ≥ 2); (e) severe systemic diseases and neuropsychiatric diseases; (f) contraindications for MRI; and (g) poorly educated (< 6 years). We also recruited 30 comorbidities- and demographically- matched healthy controls (HC). Ethical approval for this study was obtained from the Institutional Review Board of Yuncheng Central Hospital, Affiliated Hospital of Shanxi Medical University, and it is conducted as a subsidiary component of a larger multicenter investigation. All participants gave written informed consent. Detailed demographics and clinical measures are summarized in Table 1.

**Table 1:** Demographics and clinical characteristics.

Characteristic	HC (*n* = 30)	SACS (*n* = 30)	*p*
Gender (Male/Female)	23/7	23/7	0.99^a^
Age (yrs.)	65.07 ± 6.80	66.83 ± 5.56	0.28
Education (yrs.)	10.73 ± 3.23	9.57 ± 2.49	0.09
Hypertension	23/7	27/3	0.23^a^
Diabetes	5/25	7/23	0.71^a^
Hyperlipidemia	13/17	14/16	0.92^a^
Smoke	8/22	11/19	0.13^a^
Affected side	-	10 L/20R	-
MMSE	27.47 ± 0.57	27.10 ± 0.61	0.019*
MoCA	24.20 ± 1.42	23.37 ± 1.00	0.011*
Word fluency	37.03 ± 3.67	33.47 ± 6.18	0.009**
Digit symbol substitution	31.07 ± 5.25	28.10 ± 4.86	0.027*
Forwards digit-span	6.43 ± 0.94	5.63 ± 0.93	0.002**
Backwards digit-span	4.37 ± 0.76	3.77 ± 0.82	0.005**
Immediate recall	34.73 ± 6.29	30.73 ± 3.81	0.004**
Delayed recall	6.37 ± 1.22	4.60 ± 1.52	<0.001***

Data are presented as mean ± SD. MMSE, Mini-Mental State Examination; MoCA, Montreal Cognitive Assessment; RAVLT, the Rey Auditory Verbal Learning Test; a, *p*-value was obtained using the two-tailed Chi-squared test. Asterisks indicate statistical significance: *p < 0.05, **p < 0.01, ***p < 0.001.

### 2.2 Neurobehavioral assessments

Neurobehavioral assessments, including the Mini-Mental State Examination (MMSE) (38), the Montreal Cognitive Assessment (MoCA) (39) Beijing Version, the Digit Symbol Test, and the Rey Auditory Verbal Learning Test (RAVLT) (40), were administered during the days of the MRI scan. The MMSE and MoCA Beijing Version assessed global cognition. The Digit Symbol Test measured how quickly participants could pair numbers with symbols, recording the number of correct translations within 90 s. The RAVLT evaluated memory and verbal learning through a list of 15 unrelated words, which participants were asked to recall repeatedly, both immediately and after a 30-min delay. In addition, a Verbal Memory Test required participants to orally repeat increasingly longer sequences of numbers, first in forward order and then in reverse order.

### 2.3 MRI data acquisition

MRI data were collected using a Skyra 3.0 T MR scanner (Siemens, Germany). The scanning sessions included (1) T1 anatomical images (176 sagittal slices, 1-mm in-plane resolution); (2) rs-fMRI (33 axial slices, 3.8-mm slice thickness with a 0.3-mm gap, repetition time (TR) = 2 s, 240 volumes); and (3) fluid attenuated inversion recovery (FLAIR) images (TR/TE/ inversion time (TI) = 6000/396/2200 ms, voxel size 0.5 × 0.5 × 1 mm, 160 slices, 1 mm thickness). During the rs-fMRI scan, subjects were asked to remain as till as possible, keep their eyes closed, not fall asleep, and avoid thinking systematically.

### 2.4 MRI data processing

#### 2.4.1 rs-fMRI data processing

The rs-fMRI data were preprocessed using the CONN toolbox (version 20b),^1^ Statistical Parametric Mapping (SPM12),^2^ the Resting-State fMRI Data Analysis Toolkit plus (RESTplus, version 1.28),^3^ and MATLAB,^4^ following conventional methods as previously described (41). Preprocessing steps included: (1) Head motion correction: Individual scans were corrected for head motion, with functional volumes identified as outliers if head movement exceeded the 97th percentile threshold. (2) AC-PC centering and slice-timing correction: T1 anatomical images were AC-PC centered and segmented into gray matter, white matter, and cerebrospinal fluid (CSF) classes. (3) Co-registration: Functional images were co-registered to the corresponding high-resolution anatomical images and spatially normalized to Montreal Neurological Institute (MNI) space. (4) Spatial smoothing: The normalized functional images were spatially smoothed using a 6 mm full width at half maximum (FWHM) Gaussian kernel. (5) After preprocessing, smoothed data were regressed against confounding signals from white matter (5 parameters), CSF (5 parameters), motion parameters (12 parameters), and outliers detected through scrubbing. White matter and CSF signals were controlled using principal component analysis via the CompCor strategy (42).

To investigate the impact of global signal regression (GSR) (43), analyses were conducted both with and without GSR, including linear detrending. Lastly, the regressed and detrended signals were band-pass filtered using a conventional band (CB) (0.01–0.08 Hz) and five sub-frequency bands: Slow-6 (0–0.0117 Hz), Slow-5 (0.0117–0.0273 Hz), Slow-4 (0.0273–0.0742 Hz), Slow-3 (0.0742–0.1992 Hz), and Slow-2 (0.1992–0.25 Hz), as utilized in a previous study (44). Five slow-frequency bands (Slow-2 to Slow-6) were examined, following the framework proposed by Zuo et al. (17). These frequency divisions correspond to distinct physiological processes of neurovascular coupling and vascular reactivity, which may be particularly relevant to hemodynamic alterations in carotid stenosis.

#### 2.4.2 Structural data processing

VBM was performed with Statistical Parametric Mapping (SPM12) (see Footnote 2), Computational Anatomy Toolbox (CAT12.9),^5^ and MATLAB (see Footnote 4) following a conventional approach (45, 46).

The anatomical images were visually inspected and preprocessed with procedures including inhomogeneity correction, skull-stripping, registering to a diffeomorphic anatomical registration using lie algebra (DARTEL) (47) template, segmentation into GM and white matter (WM) classes, spatial normalization into the MNI space (voxel-size 1.5 mm^3^), Jacobian modulation, and spatial smoothing (a Gaussian kernel with a full width at half maximum of 8 mm).

The WMH size was calculated based on T1 and 3D T2-FLAIR images using the Lesion Segmentation Tool (LST)^6^ (48) as described in its tutorial and our recent publications (41, 45). Lesions were segmented by the lesion growth algorithm with a default initial threshold (*κ* = 0.3).

#### 2.4.3 Wavelet-based amplitude of low-frequency fluctuation (ALFF)

A continuous wavelet-transformed amplitude of low-frequency fluctuation (wavelet-ALFF) was employed to calculate the LFOs. Compared to the traditional fast Fourier transformed ALFF (FFT-ALFF), wavelet-ALFF characterizes signals in both time and frequency domains and demonstrates greater sensitivity in identifying group differences (44).

Wavelet-ALFF calculations were performed using scripts embedded in the RESTplus toolbox (49). The default mother wavelet used was db2, which has shown the highest sensitivity and reliability among five tested mother wavelets: db2, bior4.4, morl, meyr, and sym3. For more details, please refer to the original publication (44).

#### 2.4.4 Spatial parcellation of frequency-specific LFOs

To visualize the spatial distributions of dominating LFOs across the entire S2-S6 frequency bins, we employed a Z-shift approach and implemented a ‘winner-takes-all’ (WTA) strategy for parcellating the frequency-specific wavelet-ALFF topographies. This parcellation effectively illustrates the dominant spatial distributions of the LFOs, ranging from lower to higher frequencies.

#### 2.4.5 Cross-frequency analyses

To characterize the temporal interplay of different frequency bins, i.e., cross-frequency coupling, we used two algorithms: static and dynamical temporal synchronization analysis (TSA).

1. Static TSA is similar to the regional homogeneity (ReHo) (50) and measures the local LFOs synchronization among neighboring voxels at different frequency bins (i.e., S6-S2, frequency time course). This method measures the local integration of the LFOs along frequency axis.
2. Dynamical TSA extends the static TSA by capturing the temporal variability of cross-frequency synchronization across frequency bins. While static TSA provides an averaged measure of local frequency coherence, dynamical TSA focuses on the fluctuations of such coherence over the frequency domain, reflecting the dynamic integration of LFOs. To estimate this dynamic property, we applied a sliding window approach along the frequency axis. The sliding window was moved with both window size and step of two frequency bins using two types of window functions (Hamming and rectangular) to assess the robustness of temporal transitions between consecutive frequency segments (51). Within each window, TSA maps were calculated in the same way as in static TSA, representing the local cross-frequency synchronization at that frequency segment. The temporal variability of TSA was quantified as the coefficient of variation (CV = SD/mean) of the TSA values across all frequency windows, which characterizes the degree of fluctuation in the local cross-frequency synchronization pattern. Larger CV values indicate more dynamic or less stable coupling, while smaller CVs reflect more stable cross-frequency coherence patterns. The resulting TSA maps were Z-transformed and spatially smoothed with a 6-mm FWHM Gaussian kernel before group-level statistical analyses.

#### 2.4.6 The effects of gray matter atrophy

To assess the extent to which brain atrophy might account for the observed group differences, we included gray matter volume as a nuisance variable of no interest in our analysis. The results indicated that the between-group differences remained significant and largely unchanged. This finding suggests that the SACS-related differences in wavelet-ALFF are not attributable to gray matter atrophy.

#### 2.4.7 White matter lesion burden

WMH is thought to reflect impaired hemodynamics or neural activity. To investigate this, we performed voxel-wise analyses to correlate the total WMH burden with ALFF. The results revealed a significant correlation between WMH burden and ALFF in the conventional band (0.01–0.08 Hz). This relationship remained significant after controlling for age and education as nuisance variables, suggesting that neural correlates are associated with WMH burden.

### 2.5 Statistical analysis

Inter-group statistics for all clinical variables were performed using SPSS 16.0 (SPSS Inc., Chicago, IL), with a significance level set at *p* < 0.05.

For the imaging measures, independent two-sample *t*-tests were conducted to analyze ALFF. Results were corrected for multiple comparisons using cluster-level family-wise error (FWE) correction, with criteria set at voxel-level *p* < 0.001 and cluster-level *p* < 0.05. Additionally, age, gender, and head motion parameters (mean frame displacement) were controlled for in the analyses.

To investigate whether the side of stenosis influences the present findings, we further examined intergroup differences between left- and right-sided carotid stenosis relative to healthy controls. Accordingly, in our analyses, we stratified participants based on the side of stenosis. Specifically, we (1) directly compared patients with left- versus right-sided stenosis to test for potential differences; if such differences were identified, we then (2) reported group differences relative to HC separately for left- and right-sided stenosis instead of merging them; if no significant differences were observed, we (3) statistically controlled for the side of stenosis as a covariate.

To determine whether, and to what extent, the intergroup ALFF differences identified in this study are associated with cognitive performance, we used the significant clusters obtained in each frequency band (under different GSR conditions) as restricted masks. Cognitive measures that showed statistically significant between-group differences, namely MMSE, MoCA, and delayed memory scores, were entered as covariates of interest, and voxel-wise correlation analyses were conducted within these masks. A voxel-level significance threshold of *p* < 0.001, combined with a minimum cluster size of > 20 voxels, was applied to define significant correlations. This relatively stringent threshold was chosen because the masks derived from ALFF intergroup differences occupy much smaller volumes than the whole-brain search space and are therefore subject to a substantially reduced multiple-comparison burden, analogous to a small-volume correction approach.

## 3 Results

### 3.1 Participants

Thirty SACS patients and 30 controls were included in the final sample. Cognitively, SACS patients were impaired in recall memory tasks (both *ps* < 0.005), and had considerably lower global cognition (MMSE and MoCA; *ps* < 0.05), memory (memory subtest from RAVLT, forward and backward digit span; *p* = 0.005), and executive function (subtest from Digit symbol; *p* < 0.05). Pathologically, patients with SACS had significantly higher WMH loads (ratio between WMH size and total brain size, WMH volumes, and WMH number, *p* < 0.005).

### 3.2 Wavelet-based ALFF: CB

**Figure 1:**
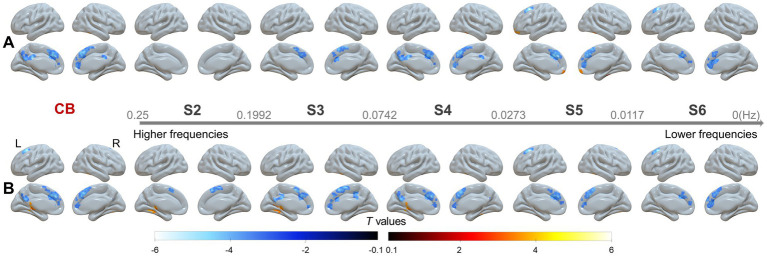
Group comparisons between the SACS patients and HC in the CB and across the five sub frequency bands **(A)** with and **(B)** without global signal regression (GSR). The results were corrected at voxel-wise *p* < 0.001 and cluster-level *p* < 0.05, FWE corrected. Hot colors indicate that SACS patients had greater wavelet-ALFF that the controls, and cold colors indicate the opposite. These maps were visualized by using the SurfIce (https://www.nitrc.org/projects/surfice).

We identified similar between-group differences both with GSR and without GSR in the conventional frequency band (CB: 0.01–0.08 Hz). Compared with HC, patients with SACS exhibited, after applying GSR, decreased wavelet-ALFF in the superior medial prefrontal cortex, anterior cingulate cortex (ACC), supplemental motor area, and precuneus/posterior cingulate cortices, along with increased wavelet-ALFF in bilateral cerebellar subregions (Figure 1; Table 2). Without GSR, SACS patients showed identical reductions in wavelet-ALFF relative to HC as those observed with GSR, whereas no significant increase were detected between groups (Figure 1; Table 2). All results were thresholded at a voxel-wise *p* < 0.001 and a cluster-level *p* < 0.05, FWE corrected.

**Table 2:** Between-group differences in wavelet-ALFF in the CB.

Contrast	Region	Extent	*t*-value	MNI coordinates		
				x	y	z
Global CB (0.01–0.08 Hz)						
Positive	Cerebellum_Crus2_L	74	4.989	−42	−75	−45
	Cerebellum_6_R	81	4.361	36	−42	−39
Negative	Frontal_Sup_Medial	367	−7.831	0	36	33
	ACC_pre_R	367	−4.617	3	45	9
	Supp_Motor_Area_R	367	−4.467	3	18	54
	Cingulate_Post	143	−5.282	0	−33	30
	Precuneus_L	143	−5.030	−3	−54	24
Noglobal CB (0.01–0.08 Hz)						
Negative	Frontal_Sup_Medial	332	−7.828	0	36	33
	Supp_Motor_Area_R	332	−4.504	6	21	54
	Frontal_Sup_2_L	64	−6.057	−24	15	54
	Precuneus_L	82	−4.889	−3	−54	24

This table shows all local maxima. x, y, and z = MNI coordinates. The results were reported with a voxel-wise *p* < 0.001 and a cluster-level *p* < 0.05, FWE corrected. BA, Brodmann’s area; *t*-value, the voxel of maximal intensity in this cluster; MNI, Montreal Neurological Institute; x, y, z, coordinates of primary peak locations in the MNI space. These results were reported with BSPMVIEW (http://www.bobspunt.com/software/bspmview/).

**Figure 2:**
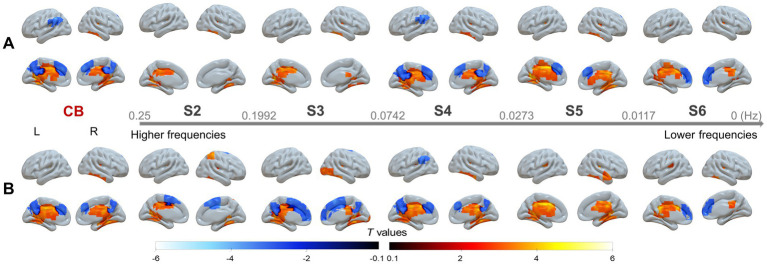
Group comparisons between patients with left-sided SACS and HC in the CB and across the five sub-frequency bands: **(A)** With and **(B)** without global signal regression (GSR). The results were corrected at voxel-wise *p* < 0.001 and cluster-level *p* < 0.05, FWE corrected. Hot colors indicate that SACS patients had greater wavelet-ALFF that the controls, and cold colors indicate the opposite. These maps were visualized by using the SurfIce (https://www.nitrc.org/projects/surfice).

**Figure 3:**
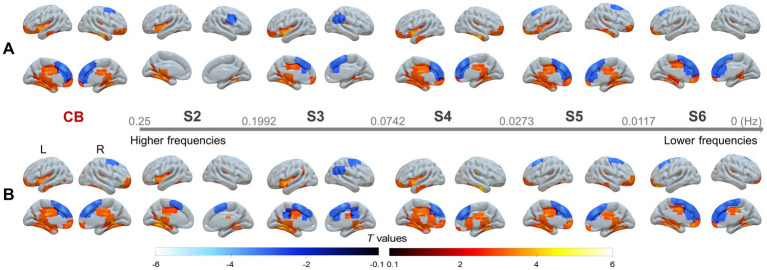
Group comparisons between patients with right-sided SACS and HC in the CB and across the five sub-frequency bands: **(A)** With and **(B)** without global signal regression (GSR). The results were corrected at voxel-wise *p* < 0.001 and cluster-level *p* < 0.05, FWE corrected. Hot colors indicate that SACS patients had greater wavelet-ALFF that the controls, and cold colors indicate the opposite. These maps were visualized by using the Surf Ice (https://www.nitrc.org/projects/surfice).

In addition, although no significant differences were identified between patients with left- and right-sided stenosis, significant between-group differences were observed in each subgroup, namely for left-sided stenosis versus HC (Figure 2) and for right-sided stenosis versus HC (Figure 3). These findings were largely consistent with those obtained from the pooled analysis.

### 3.3 Wavelet-based ALFF: sub-frequency bands

We next analyzed between-group differences across progressively higher frequency bands, from S6 (0–0.0117 Hz) to S2 (0.1992–0.25 Hz) (Figure 1; Table 3). Overall, these results largely replicated those observed in the CB, though the significant clusters exhibited a larger spatial extent.

**Table 3:** Between-group differences in wavelet-ALFF across sub-frequency bands (with global signal regression).

Contrast	Region	Extent	*t*-value	MNI coordinates		
				x	y	z
Global S6 (0–0.0117 Hz)						
Negative	Frontal_Sup_2_L	69	−6.611	−24	15	54
	Frontal_Sup_Medial	77	−5.456	0	33	36
	ACC_pre_L	152	−4.859	−3	45	15
	ACC_sup_R	152	−4.15	6	24	21
Global S5 (0.0117–0.0273 Hz)						
Positive	Fusiform_R	111	4.761	45	−54	−24
	OFCant_L	61	4.57	−24	51	−15
Negative	Frontal_Sup_Medial	387	−7.39	0	36	33
	Supp_Motor_Area_R	387	−5.497	3	18	54
	ACC_pre_R	387	−4.644	6	45	9
	Frontal_Sup_2_L	98	−6.366	−24	15	54
Global S4 (0.0273–0.0742 Hz)						
Positive	Cerebellum_Crus2_L	80	5.003	−42	−75	−45
	Cerebellum_9_L	80	4.03	−18	−51	−57
	Cerebellum_8_R	76	4.138	39	−45	−48
Negative	Frontal_Sup_Medial	215	−7.327	0	36	33
	Supp_Motor_Area_L	215	−4.277	−3	18	48
	Precuneus_L	151	−5.167	−3	−54	24
	Cingulate_Post_L	151	−4.983	0	−33	30
	ACC_pre_R	92	−4.645	3	45	9
Global S3 (0.742–0.1992 Hz)						
Positive	Cerebellum_7b_L	113	6.094	−39	−66	−54
	Cerebellum_Crus2_L	113	4.277	−36	−81	−36
	Cerebellum_8_R	68	5.684	30	−60	−57
Negative	Supp_Motor_Area	89	−6.875	0	6	54
	Frontal_Sup_Medial_L	80	−4.994	−3	27	36
	Frontal_Mid_2_R	29	−4.788	45	30	30
	Cingulate_Post_L	28	−4.435	−3	−33	30
Global S2 (0.1992–0.25 Hz)						
Positive	Cerebellum_7b_L	68	5.552	−39	−66	−54

This table shows all local maxima. x, y, and z = MNI coordinates. The results were reported with a voxel-wise *p* < 0.001 and a cluster-level *p* < 0.05, FWE corrected. BA, Brodmann’s area; *t*-value, the voxel of maximal intensity in this cluster; MNI, Montreal Neurological Institute; x, y, z, coordinates of primary peak locations in the MNI space. These results were reported with BSPMVIEW (http://www.bobspunt.com/software/bspmview/).

We first report the findings with GSR. In S6, reduced wavelet-ALFF was observed in the left superior frontal gyrus, superior medial frontal gyrus, and ACC. In S5 and S4, additional reductions were found in the SMA and left precuneus/posterior cingulate cortex (PCC), together with higher wavelet-ALFF in the right fusiform and bilateral OFC. In S3, lower wavelet-ALFF appeared in the SMA, left superior medial frontal gyrus, right middle frontal gyrus, and left PCC, while higher wavelet-ALFF was evident in bilateral cerebellar subregions. In contrast, in S2, only higher wavelet-ALFF was detected in the left Cerebellum_7b.

Notably, these frequency-dependent alterations exhibited a spatial–temporal hierarchy: clusters in the anterior midline (ACC and superior medial frontal) remained significant from the lowest frequency band (S6) up to S3, whereas those in the posterior midline regions (PCC/precuneus) persisted mainly at the higher frequencies (S4 and S3). Similar patterns were observed when analyses were conducted without GSR (Figure 1; Table 4).

**Table 4:** Between-group differences in wavelet-ALFF across sub-frequency bands (without global signal regression).

Contrast	Region	Extent	*t*-value	MNI Coordinates		
				x	y	z
Nonglobal S6 (0–0.0117 Hz)						
Negative	Frontal_Sup_2_L	82	−6.301	−24	15	54
	Frontal_Sup_Medial_R	255	−5.24	3	57	21
	Supp_Motor_Area_R	255	−3.932	3	21	54
Nonglobal S5 (0.0117–0.0273 Hz)						
Positive	Cerebellum_6_R	219	5.28	42	−57	−27
	Vermis_6	103	4.878	0	−60	−21
Negative	Frontal_Sup_2_L	92	−6.512	−24	15	54
	Frontal_Sup_Medial_L	286	−6.409	−3	36	33
	Supp_Motor_Area_R	286	−4.912	3	18	54
Nonglobal S4 (0.0273–0.0742 Hz)						
Positive	Vermis_6	140	5.848	3	−66	−24
	Hippocampus_L	68	5.049	−24	−36	0
	Cerebellum_Crus1_R	128	4.965	45	−54	−30
	Cerebellum_8_R	128	4.659	27	−45	−48
Negative	Frontal_Sup_Medial	185	−7.482	0	36	33
	Supp_Motor_Area_L	185	−4.03	−3	21	54
	Precuneus_L	103	−4.974	−3	−54	24
	Frontal_Sup_Medial	89	−4.616	0	54	24
	ACC_pre	89	−4.257	0	51	3
Nonglobal S3 (0.742–0.1992 Hz)						
Positive	Cerebellum_Crus2_L	114	5.626	−36	−81	−36
	Cerebellum_7b_L	114	5.398	−39	−66	−54
	Hippocampus_L	142	5.509	−33	−39	−3
	Thal_PuM_L	142	4.727	−12	−33	6
	Cerebellum_6_R	76	4.193	36	−39	−36
Negative	Supp_Motor_Area	143	−7.025	0	6	54
	Frontal_Sup_Medial_L	104	−5.626	−3	27	36
	Cingulate_Post_L	96	−4.737	−3	−33	30
	Precuneus_L	96	−4.381	−6	−57	21
	Frontal_Sup_Medial_L	71	−4.452	−3	51	3
Nonglobal S2 (0.1992–0.25 Hz)						
Positive	Hippocampus_L	115	5.598	−21	−36	3
	Cerebellum_Crus2_L	82	5.358	−39	−78	−36
	Cerebellum_7b_L	82	4.984	−39	−66	−54
Negative	Supp_Motor_Area_R	84	−5.096	6	21	54

This table shows all local maxima. x, y, and z = MNI coordinates. The results were reported with a voxel-wise *p* < 0.001 and a cluster-level *p* < 0.05, FWE corrected. BA, Brodmann’s area; *t*-value, the voxel of maximal intensity in this cluster; MNI, Montreal Neurological Institute; x, y, z, coordinates of primary peak locations in the MNI space. These results were reported with BSPMVIEW (http://www.bobspunt.com/software/bspmview/).

### 3.4 Spatial hierarchy of frequency-specific wavelet-ALFF

**Figure 4:**
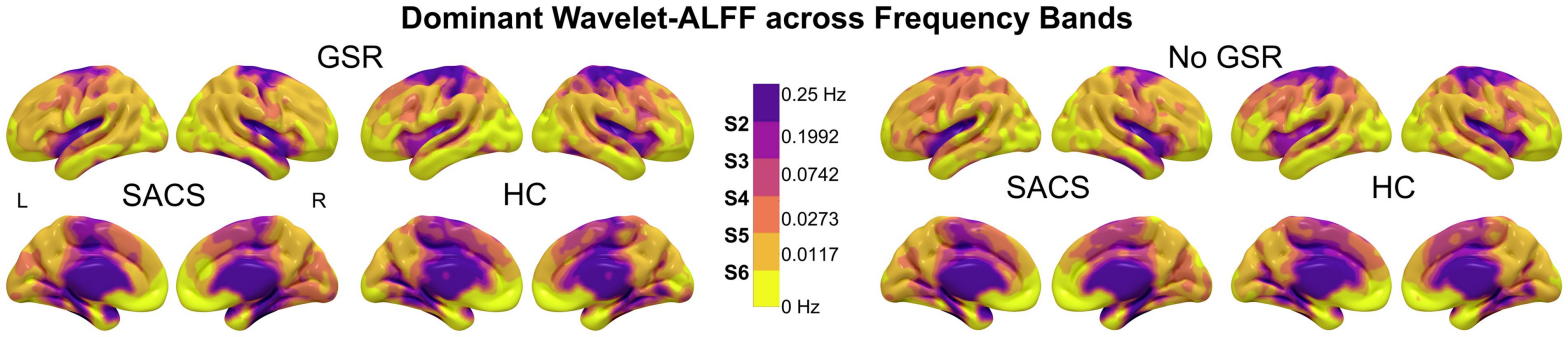
Dominant wavelet-ALFF distributions across the frequency bands. Using a ‘winner-takes-all’ strategy, the frequency band specific ALFF distributions are outlined and parcellated. GSR, global signal regression; SACS, severe asymptomatic carotid stenosis; HC, healthy controls; L, left; R, right. These maps were visualized by using the Surf Ice (https://www.nitrc.org/projects/surfice).

The group-averaged wavelet-ALFF is shown in Supplementary Figure S1. The spatial distribution of wavelet-ALFF exhibits a cortical hierarchical pattern along the frequency axis. From lower to higher frequencies, wavelet-ALFF gradually decreases in association cortices (particularly in the medial prefrontal and PCC regions) while it progressively increases in sensory/somatomotor and subcortical regions. Using the WTA strategy, we parcellated and identified that across the entire frequence range (0–0.25 Hz), from lower (S6: 0–0.0117 Hz) to higher (S2: 0.1992–0.25 Hz) frequencies, the dominating wavelet-ALFF showed hierarchical organization along a posterior-to-anterior and association-to-primary/subcortical axis. Overall, this hierarchical pattern was consistent within the HC group but showed divergence in SACS patients, particularly within the DMN. The results were not significantly affected by GSR (Figure 4).

### 3.5 Temporal modulation across the frequencies

**Figure 5:**
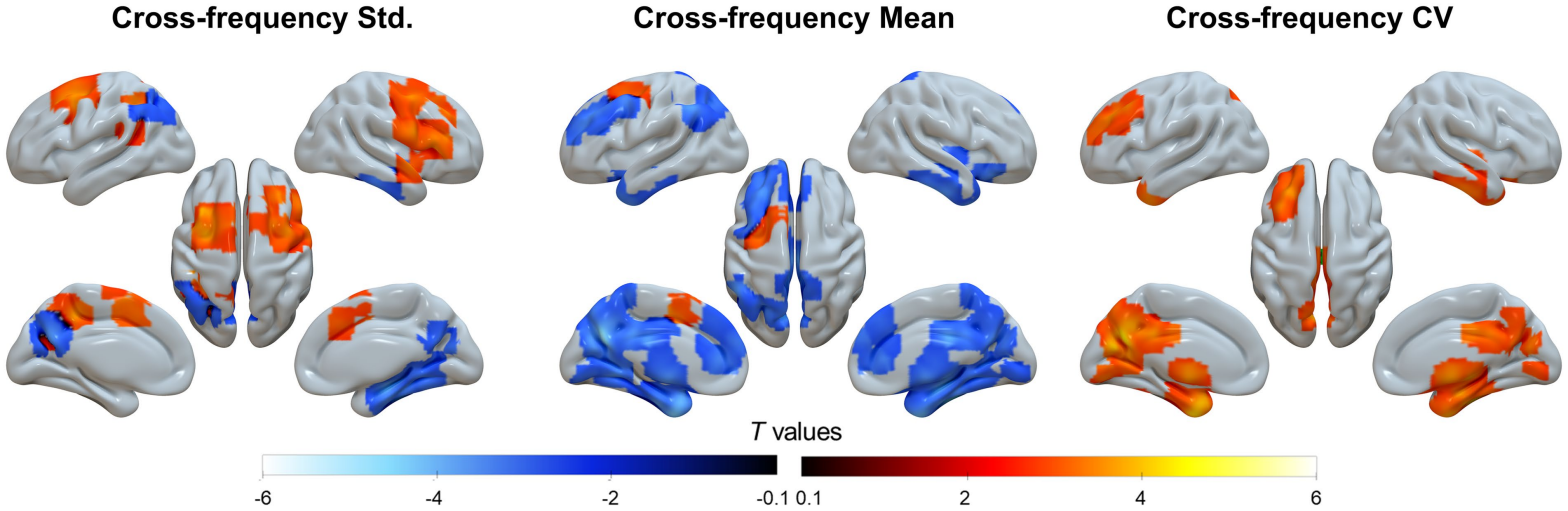
Cross-frequency analyses. The cross-frequency coupling includes three analyses from left to right: static temporal synchronization analysis (TSA), mean of dynamic TSA using the Hamming sliding-window, and temporal variation (coefficients of variation, CV) of dynamic TSA using the Hamming sliding-window. These maps were visualized by using the Surf Ice (https://www.nitrc.org/projects/surfice).

For the static, cross sliding-window average, and temporal variation TSA analyses, group-level results indicated that the DMN and associative cortices exhibited the highest cross-frequency homogeneity but the lowest frequency variability, whereas the primary sensorimotor and subcortical regions showed the opposite pattern (Supplementary Figure S2). The TSA results further revealed that patients with SACS showed lower regional cross-frequency synchronization in precuneus/PCC but higher synchronization within the salience and somatomotor systems (Figure 5, left panel).

When applying a Hamming window with both window length and step size of two frequency bins, the mean regional cross-frequency synchronization was prominently attenuated (Figure 5, middle panel), whereas the coefficient of variation is significantly enhanced in the same topographies (Figure 5, right panel), suggesting greater temporal variability across the frequency bands.

### 3.6 The effects of gray matter atrophy

After controlling for voxel-wise gray matter volume as a nuisance covariate, the between-group effects remained significant and largely unchanged, suggesting that the observed differences in frequency-specific wavelet-ALFF could not be attributed to gray matter atrophy.

### 3.7 The effects of WMH loads

**Figure 6:**
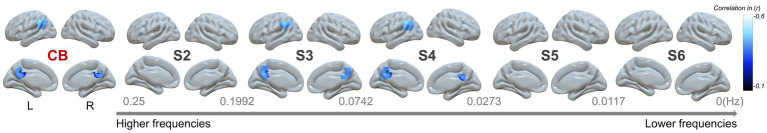
Whole brain voxel-wise regression between WMH load and regional ALFF. The results were corrected at voxel-wise *p* < 0.001 and cluster-level *p* < 0.05, FWE corrected. The blue colorbar indicates the degree of negative correlation between WMH load and ALFF, and the clusters represent regions showing significant negative correlations after thresholding. These maps were visualized by using the Surf Ice (https://www.nitrc.org/projects/surfice).

Voxel-wise regression analyses between WMH load and wavelet-ALFF revealed that WMH burden was negatively correlated with LFOs in association cortices, particularly within the DMN. These results suggest that neurolesional effects follow a cortical reallocation pattern, whereby greater WMH load is associated with reduced LFOs in higher-order association regions (Figure 6). This reorganization may partly explain why aging-related cognitive decline is linked to decreased intrinsic activity in the DMN and other association cortices.

### 3.8 Association analyses

**Figure 7:**
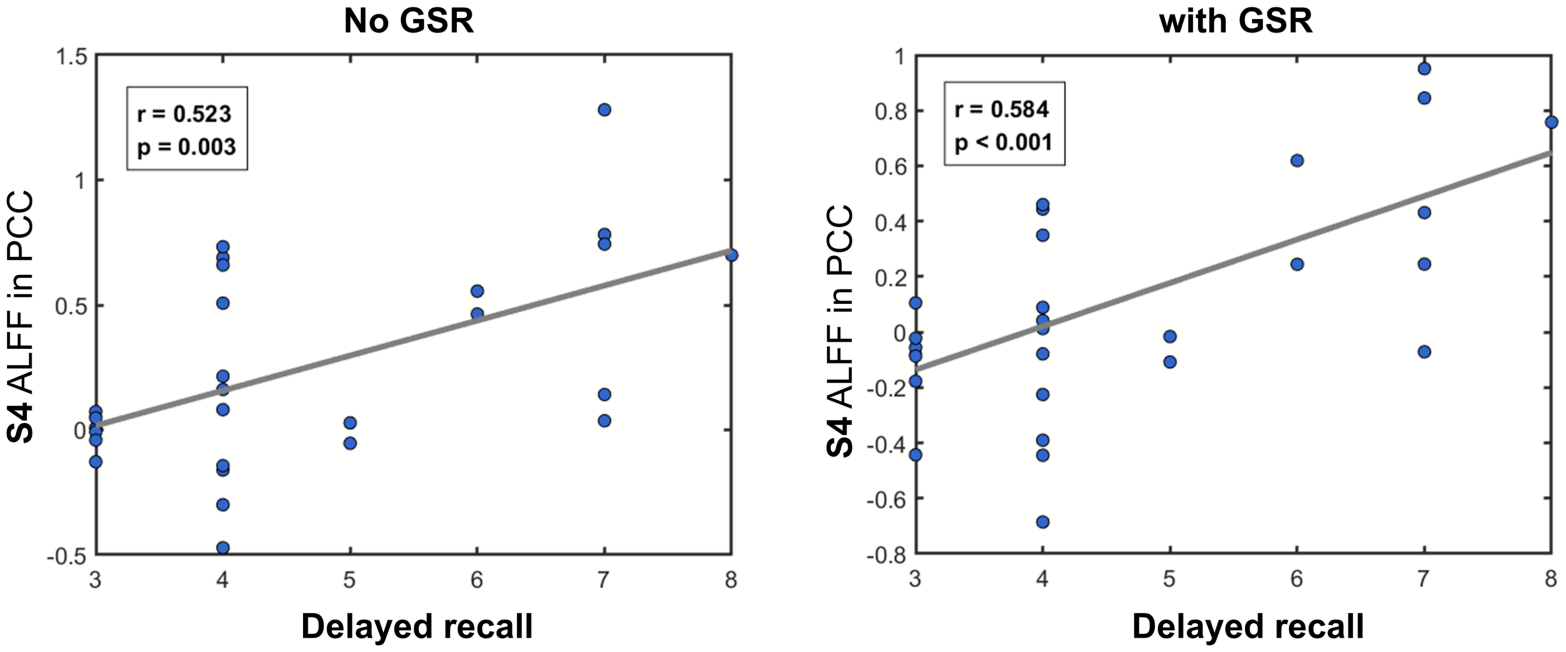
Correlation analyses. The scatter plots illustrate the association between delayed recall memory and ALFF in the PCC region that exhibits intergroup differences, under both GSR and no GSR conditions.

In SACS patients, delayed memory (RAVLT sub-score) demonstrated significant correlations with ALFF in the PCC within the S4 frequency band. This correlation was observed as significant positive relationships under both no GSR (*r* = 0.523, *p* = 0.003) and with GSR (*r* = 0.584, *p* < 0.001) conditions (Figure 7). These findings suggest that the decline in delayed recall performance parallels the reduction in ALFF within the PCC region.

## 4 Discussion

We investigated the spatiotemporal frequency structure of resting-state brain activity using wavelet-based ALFF. Patients with SACS exhibited frequency-specific alterations and abnormal cross-frequency coupling of LFOs, which generally followed a cortical hierarchy from lower to higher frequencies. These findings suggest that cognitive impairments in SACS may arise from disrupted cross-frequency interactions within the DMN. More broadly, this framework may provide new insights into the mechanisms underlying other major brain diseases and related physiological processes.

One key finding concerns the spatial organization of the dominant LFOs. Using the WTA, we identified that the dominant LFOs measured by wavelet-ALFF follow a spatial axis extending from posterior to anterior regions and from association to primary/subcortical areas, with frequencies progressing from lower (S6: 0–0.0117 Hz) to higher (S2: 0.1992–0.25 Hz) bands. This pattern aligns with recent magnetoencephalography findings and converges with observations of functional connectivity gradients, anatomical variability, and semantic category processing (10, 13–17), suggesting a fundamental principle of functional organization in the human brain. Patients with SACS displayed distinct spatial patterns of LFOs in the cortical midline and lateral prefrontal regions. Notably, alterations within the DMN showed a frontal-to-posterior and anterior-to-rostral gradient with increasing frequency. These results are consistent with previous reports in Alzheimer’s disease and mild cognitive impairment (18, 52), suggesting that abnormal frequency-dependent LFOs in the DMN may be a common feature underlying cognitive dysfunction across disorders.

Contrary to our hypothesis and previous reports, dominant LFOs were observed in the primary visual cortex at medium-low frequencies. This unexpected finding may partly reflect the relatively coarse frequency sampling (S2–S6), which could have blurred distinctions between medium and low frequency bands. Alternatively, it may point to the complex dynamics of resting-state fMRI time series in the visual stream. For example, evidence from human brain connectomics and graph-theoretical analyses suggests that the visual cortex serves versatile roles as a central hub within large-scale brain networks (53). These results imply that reorganization of spatially dominant LFOs and alterations in cross-frequency temporal coupling and propagation warrant further exploration. Given recent research emphasizing the temporal dynamics of frequency processing as a fundamental aspect of brain function (10, 14), this points to the need for a deeper examination of cross-frequency coupling changes.

### 4.1 Cross-frequency coupling

The second key finding of this study is temporal synchronization of LFOs along the frequency bands. Remarkably, the static TSA is the highest in the DMN and subcortical regions but lower in sensorimotor and lateral temporal regions. This pattern, however, is reversed in the dynamic sliding-window analysis: the DMN and association cortex have higher temporal variability, but sensorimotor and subcortical regions have less temporal variability along the frequency axis. This topographic arrangement nicely aligns with the spatial parcellation, and is also closely related to functional connectivity gradients (14), anatomical variability (54), myeloarchitecture (55), semantic category-processing (56), and cortical expansion (57), demonstrating a functional organization of the intrinsic brain activity.

While the DMN has been considered to be at the apex of human brain functional hierarchy, involving in non-environment-driven real-time tasks such as abstract cognition (11), increasing evidence suggests that the DMN may possess a repertoire of functional roles that support most brain functions (21). Our current finding is consistent with this notion: that is, the DMN processes a broad range of frequencies, and has higher cross-frequency coupling and maintains lower temporal variability. Additionally, the subcortical regions have been seen as generators of rhythmicity (58), of which the frequency characteristics are still largely unclear.

SACS patients exhibited decreased static DMN cross-frequency coupling, and increased cross-frequency coupling in the somatomotor and subcortical regions. This double disassociation suggests abnormal cross-frequency synchronization in the DMN and somatomotor networks—two networks that have been suggested in the pathological process of SACS. Indeed, SACS is associated with hemispheric hemodynamic alteration. This alteration may lead to prolonged hemodynamic latency and silent lesions (59, 60). The latter two significantly affect the time-frequency components of intrinsic brain activity.

Additionally, SACS patients showed enhanced temporal variability of LFOs along the frequency axis, suggesting that LFOs in the DMN have abnormal cross-frequency temporal synchronization. This abnormality may reflect the impairment of DMN functionality related to cognitive impairment, since the important roles of DMN on cognitive function have been established (11, 12, 21). One possibility is that the DMN, as a fundamental structure of advanced cognitive function, requires high stability to maintain self-function---serving advanced cognitive behaviors. Instability of the DMN may distract the ability to process advanced cognitive functions and segregate itself from other large-scale networks, such as the salient and sensorimotor networks. Nevertheless, it is still unclear how the changes in the time-frequency structure of the DMN LFOs underly the basis of cognitive impairment, and further research is needed to elaborate this issue in the future.

### 4.2 The effects of brain atrophy and lesion burden

Between-group comparisons of wavelet-ALFF did not significantly change when controlling for individual voxel-wise gray matter volume, suggesting that the identified group differences cannot be fully explained by the brain atrophy. This finding is consistent with prior report in MCI (18).

WMH burden was negatively correlated with LFOs in the medial prefrontal cortex across several frequency bands, especially in the S6. This finding suggests that WMH is associated with reduced intrinsic brain activity. Pathologically, WMH represents structural dysconnectivity and impaired hemodynamics, and the latter two have been related to abnormal resting-state brain activity. In an exploratory analysis, we found that the control of WMH could not fully offset the between-group differences, suggesting that the between-group differences in wavelet-ALFF is a characteristic alteration. We then correlated the WMH burden with ALFF, and found the WMH load was associated with overall wavelet-ALFF shift, and the WMH load was significantly correlated with ALFF in higher frequencies, which indicates vascular components in higher frequencies.

In the correlation analysis, we identified a significant association only between delayed recall memory and region-specific ALFF in the PCC, both with and without GSR. On the one hand, we did not observe significant correlations with other cognitive measures, including the global cognitive indices MMSE and MoCA. This may reflect the weaker neuroanatomical specificity of these global scores, or it may be related to variations in the analyzable mask size across different frequency bands and GSR conditions, which could have reduced our statistical power. On the other hand, delayed recall, as an aspect of semantic memory, may be the cognitive domain most vulnerable to ICA hypoperfusion, particularly given its strong association with the PCC, a core node of the DMN. This pattern suggests a functional neuroanatomical basis for semantic memory impairment. Moreover, recent large-cohort studies have similarly highlighted that carotid artery stenosis has a pronounced impact on semantic memory (61).

Several limitations should be acknowledged in the present study. First, the frequency analysis employed pre-defined frequency bins (i.e., CB, S2-S6) of uneven lengths, which may limit its ability to detect architectural attributes and between-group differences. Second, while this study examined wavelet-ALFF at the whole brain level, analyzing it at the cortical level may provide more meaningful insights. Although non-invasive functional neuroimaging has advanced our understanding of cortical organization, subcortical organization remains poorly characterized. Consequently, this study does not fully explain why the GSR procedure has a more pronounced effect on results from the subcortex and cerebellum. Future research that separately analyzes cortical and subcortical structures could enhance our understanding of subcortical organization.

Although the five frequency bands were adopted from prior literature, they align with known oscillatory components of BOLD activity. Specifically, the lower-frequency ranges (Slow-4 and Slow-5) are closely associated with vascular dynamics and are thus hypothesized to be more affected by hypoperfusion or delayed hemodynamics in carotid stenosis. Given that chronic hypoperfusion is expected to alter neurovascular coupling and delay hemodynamic responses, frequency components within the lower bands (Slow-4 and Slow-5) may be particularly sensitive to vascular compliance and flow regulation abnormalities. Higher bands (Slow-2 and Slow-3) might capture faster neural oscillatory influences that could also be disrupted under impaired perfusion.

In addition, we note the potential influence of sex on the observed between-group differences. Our sample included a relatively larger proportion of male participants; although the patient and HC groups were matched on sex and age (as well as education level and chronic comorbidities), how sex may modulate ALFF alterations remains not fully clear. We therefore conducted sex-stratified between-group analyses. In the male subsample (patients vs. healthy controls), the between-group differences were consistent with the main findings, supporting the robustness of our results. By contrast, given the small number of female participants, female-only analyses were likely underpowered; our exploratory results showed a similar spatial distribution to the overall and male-specific patterns, but with weaker effects that did not survive multiple comparison correction. Accordingly, while including sex as a covariate in the primary model likely provides a reasonable estimate of the overall group effect, potential sex-specific differences remain an open question that should be examined in future studies with larger, adequately powered samples.

## 5 Conclusion

We identified unique frequency structure of the DMN LFOs using wavelet-ALFF, and found characteristic alterations underlying cognitive impairment in SACS. Our data suggested that frequency specific changes underlying cognitive impairment in SACS associate with abnormal cross-frequency spatial rearrangement and temporal coupling. These findings may provide new insights into the understanding of other major brain diseases and physiological processes.

## Data Availability

The original contributions presented in the study are included in the article/Supplementary material, further inquiries can be directed to the corresponding authors.

## Footnotes

1
https://web.conn-toolbox.org/

2
http://www.fil.ion.ucl.ac.uk

3
http://www.restfmri.net/forum/

4
https://www.mathworks.com

5
http://dbm.neuro.uni-jena.de/cat

6
www.applied-statistics.de/lst
