# Supplementary material for: Altered frequency architecture of spontaneous brain activity in asymptomatic carotid stenosis: a wavelet-based resting-state fMRI study
Source: Front Neurol. 2026 Jan 22;17:1683526. doi: 10.3389/fneur.2026.1683526 (PMC12872527; doi:10.3389/fneur.2026.1683526)
Supplement: Supplementary file 1 [file Table_1.DOCX]

Supplementary Material

# Supplementary Results


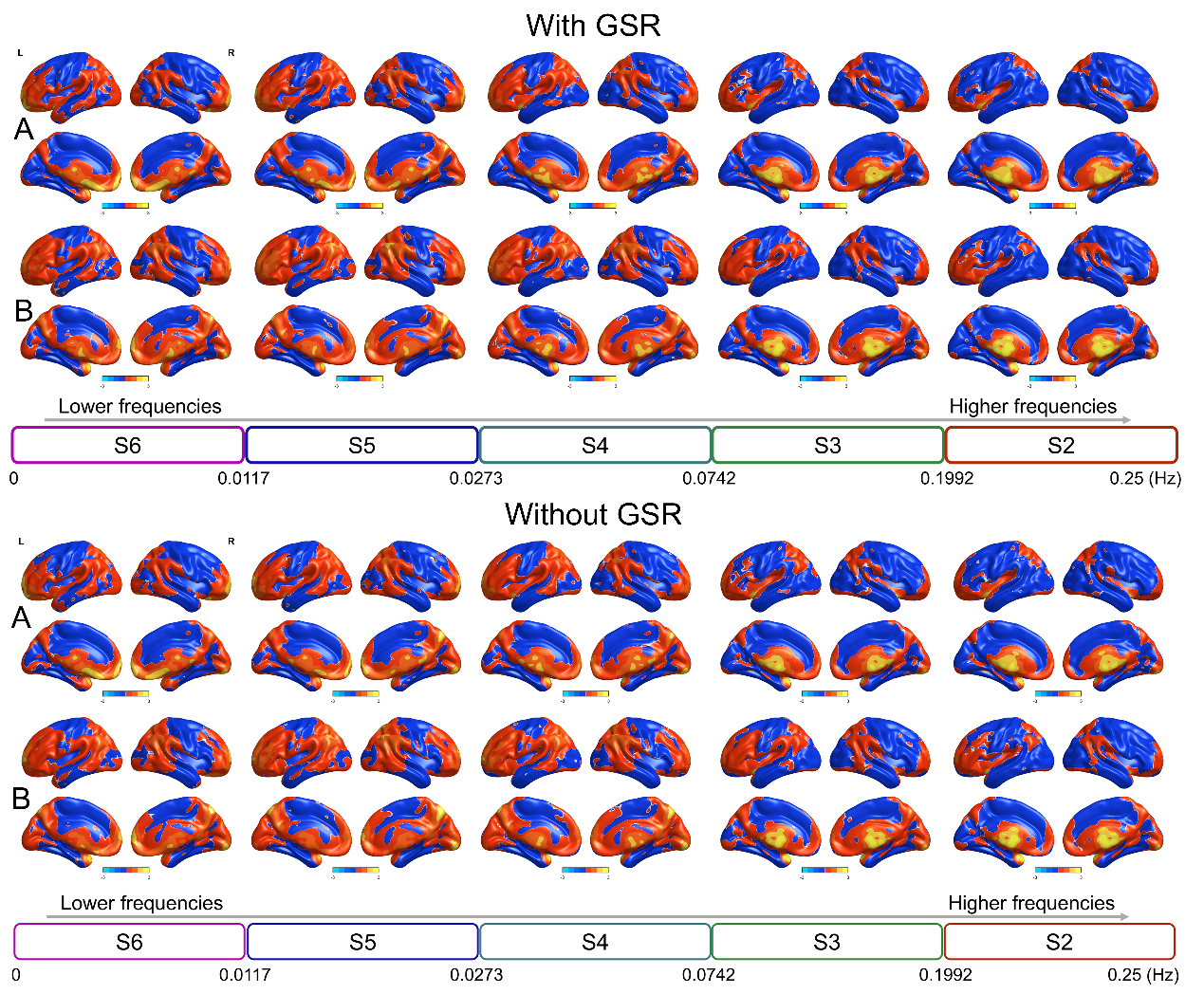


**Fig. S1.** **Group-average wavelet-ALFF across frequency bands**. The upper panel shows group average wavelet-ALFF with global signal regression (GSR), and the lower panel without GSR, both for SACS patients (A) and healthy controls (HC). These maps were visualized by using the BrainNet viewer (<https://www.nitrc.org/projects/bnv/>).


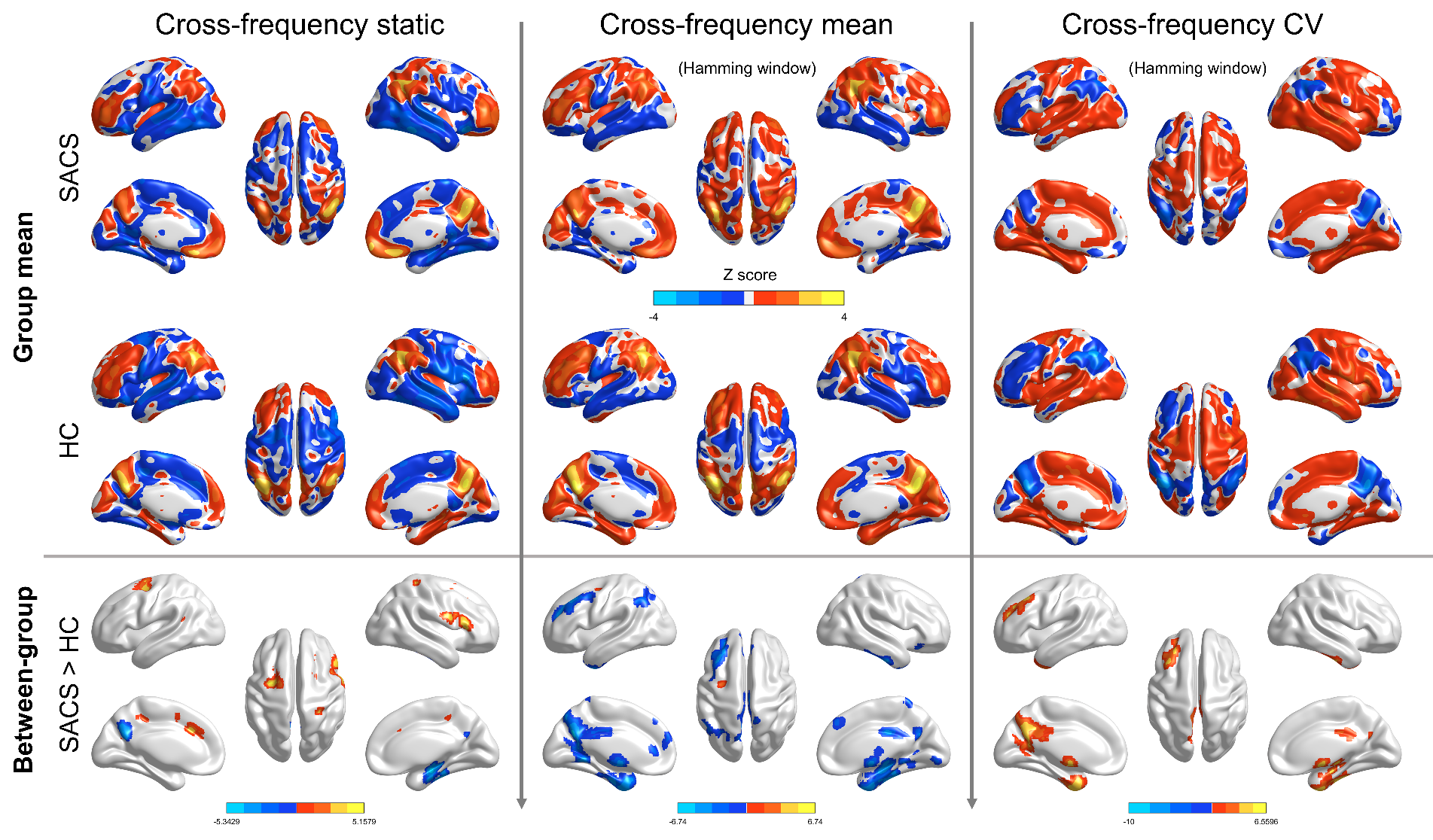


**Fig. S2. Cross-frequency analyses.** The cross-frequency coupling includes three analyses from left to right: static temporal synchronization analysis (TSA), mean of dynamic TSA using the Hamming sliding-window, and temporal variation (coefficients of variation, CV) of dynamic TSA using the Hamming sliding-window. Group mean for SACS patients (upper panel) and healthy controls (middle panel), and their between-group comparisons (lower panel). These maps were visualized by using the BrainNet viewer (<https://www.nitrc.org/projects/bnv/>).
